# Clinical and Molecular Characterization of Xia–Gibbs Syndrome: Expanding the Phenotypic Spectrum in a Brazilian Cohort

**DOI:** 10.1111/cge.14777

**Published:** 2025-06-11

**Authors:** Maísa Ganz Sanchez Sennes, Laura Machado Lara Carvalho, Matheus Augusto Araújo Castro, Giovana Manilli Toccoli, Sofia de Oliveira Farias, Davi Mendes Campo Fialho, Eny Maria Goloni Bertollo, Erika Cristina Pavarino, Larissa Sampaio de Athayde, Cecilia Barbosa Buck, Maria Betânia Pereira Toralles, Maria Isabel Melaragno, Mariluce Riegel‐Giugliani, Gustavo Marquezani Spolador, Paulo Alberto Otto, Caroline Brandão Piai, Fernando Kok, Ceres Schmitz Cechella, Carla Rosenberg, Juan Clinton Llerena, Débora Romeo Bertola, Salmo Raskin, Chong Ae Kim, Ana Cristina Victorino Krepischi

**Affiliations:** ^1^ Institute of Biosciences, Department of Genetics and Evolutionary Biology Human Genome and Stem Cell Research Center, University of Sao Paulo São Paulo Brazil; ^2^ Department of Morphology and Genetics Federal University of Sao Paulo São Paulo Brazil; ^3^ Faculty of Medicine of Sao José do Rio Preto São José do Rio Preto Brazil; ^4^ Universidade de São Paulo, Faculdade de Medicina, Departamento de Neurologia Mendelics Genomic Analysis São Paulo Brazil; ^5^ Anhembi Morumbi University Piracicaba Brazil; ^6^ Faculty of Medicine, Federal University of Bahia Salvador Brazil; ^7^ Casa dos Raros, Center for Comprehensive Care and Training in Rare Diseases Anthony Daher Laboratory, Casa dos Raros Porto Alegre Brazil; ^8^ Unit of Pediatric Pain and Palliative Care Instituto da Criança, Clinics Hospital of University of Sao Paulo Faculty of Medicine São Paulo Brazil; ^9^ Genetics Unit Instituto da Criança, Hospital das Clínicas, Faculdade de Medicina da Universidade de São Paulo São Paulo Brazil; ^10^ Cechella Medical Clinic Novo Hamburgo Brazil; ^11^ National Institute Fernandes Figueira ‐ Fiocruz Rio de Janeiro Brazil; ^12^ Postgraduate Program in Child and Adolescent, Department of Pediatrics Federal University of Paraná Curitiba Brazil

**Keywords:** *AHDC1*, developmental regression, gonadal alterations, hyperphagia, phenotypic spectrum, shortening of the Achilles tendon, Xia–Gibbs syndrome

## Abstract

Xia–Gibbs syndrome (XGS) is a rare intellectual disability (ID) syndrome caused by *de novo AHDC1* pathogenic variants. We characterized clinical and molecular features of 16 Brazilian patients with XGS. Patient data were collected through semistructured interviews with family members, reanalysis of previous health and genetic assessments, and clinical reports from physicians. Genomic variants and their segregation were validated via Sanger sequencing. Statistical analyses were conducted to evaluate genotype–phenotype associations. Twelve novel *AHDC1* causative variants were documented. ID, hypotonia, motor developmental delay, and varied nonspecific facial dysmorphisms were observed in all patients, while speech impairment and autism spectrum disorder were present in nearly all. Three frequent phenotypes, not previously reported, were identified: hyperphagia/food obsession, genital/gonadal alterations in males, and shortening of the Achilles tendon. Additionally, our findings provide statistically significant support for previously reported genotype–phenotype associations between pathogenic variants in the first half of the *AHDC1* coding region and the occurrence of epilepsy and scoliosis. We also propose a novel association between N‐terminal variants and developmental regression. In summary, our results broaden the clinical phenotype of XGS, with musculoskeletal and genital/gonadal abnormalities highlighting the multisystem involvement in this condition, beyond neurodevelopmental deficits. Comprehensive phenotypic assessments in all identified XGS cases are recommended to accurately recognize and associate novel clinical signs with XGS.

## Graphical abstract

We present a clinical and molecular characterization of 16 Brazilian individuals with Xia–Gibbs syndrome, identifying 12 novel *AHDC1* variants and four phenotypes not previously associated. Our findings support existing genotype–phenotype associations and suggest a new relation, expanding the known phenotypic and genetic spectrum of the syndrome.

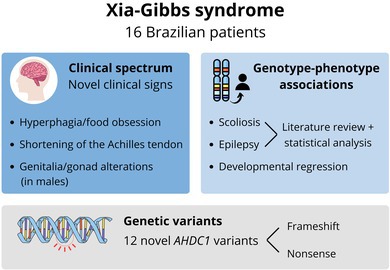

## 1 Introduction

Xia–Gibbs syndrome (XGS, OMIM#615829) is an autosomal dominant syndromic form of intellectual disability (ID) [1] caused by heterozygous *de novo AHDC1* pathogenic variants. XGS was first described by Xia and colleagues in 2014, with the identification of *de novo* indel *AHDC1* variants in four unrelated individuals with syndromic ID [2]. Subsequent publications also reported patients with XGS carrying nonsense *AHDC1* variants [3, 4, 5, 6]. Missense variants [7], as well as deletions and duplications comprising *AHDC1*, have also been reported, with clinical signs overlapping with XGS [8, 9, 10, 11, 12].

Accurate prevalence data for XGS are not available, partly due to underdiagnosis, but the condition is considered rare. The overall frequency of ID is estimated at 10.37 per 1000 population (95% CI: 9.55–11.18), based on a meta‐analysis of 52 studies [13]. In a cohort of 2157 patients with ID and/or developmental delay, pathogenic variants in *AHDC1* were identified in only seven individuals (0.32%) [3]. Furthermore, the Xia–Gibbs Society currently documents approximately 530 diagnosed individuals worldwide, while around 97 cases have been reported in the literature (including frameshift, nonsense and missense variants, but excluding microdeletions and microduplications involving *AHDC1*) [14].

Increasing evidence suggests the presence of phenotypic variability and a broader clinical spectrum. Clinically, XGS is characterized by variable degrees of ID, and several other clinical signs may be present, including hypotonia, motor development delay, absent or impaired speech, structural brain abnormalities, and various nonspecific mild dysmorphisms. Additional relatively recurrent features include scoliosis, ataxia, epilepsy, obstructive sleep apnea, aggressiveness, self‐harm, anxiety, low social interaction, attention‐deficit/hyperactivity disorder, and autism spectrum disorder (ASD) [1].

In the literature, most reported cases are from North America or Europe. Nevertheless, data from other continents, such as South America, are very limited [6, 15, 16, 17]. We aimed to contribute to broadening the phenotypic spectrum of this condition by presenting a thorough clinical and molecular portrait of 16 XGS cases from a Brazilian cohort. We reported 12 novel causative variants and suggested a novel genotype–phenotype association for developmental regression, as well as three characteristics not previously described as frequent in XGS: hyperphagia/food obsession, shortening of the Achilles tendon, and gonadal/genital anomalies in males.

## 2 Patients and Methods

We recruited 16 Brazilian individuals previously diagnosed with XGS through exome sequencing, with pathogenic/likely pathogenic variants classified according to the American College of Medical Genetics and Genomics guidelines [18]. The patients' ages varied from 4 to 26 years, with equal numbers of males and females.

The Research Ethics Committee of the Institute of Biosciences (University of São Paulo—Brazil) approved this study (CAAE 80921117.5.00000.5464). A legal guardian for each patient signed an informed consent term authorizing the collection of clinical and molecular data, as well as blood samples and the use of photographs in scientific publications.

Clinical and molecular data were documented via the following strategies: (a) semistructured interviews (script provided in Supporting Information 1) applied to at least one parent of each patient; (b) recovery of the results of previously performed health and genetics tests; and (c) contact with physicians and other health professionals who assist the patients.

Data from the present study and those described by Khayat et al. [7] were combined to compare the frequencies of scoliosis and epilepsy among patients carrying N‐terminal and C‐terminal variants. Statistical analysis of the genotype–phenotype associations was conducted according to the method described by Pardono et al. [19]. This approach allows the comparison of different datasets and accounts for cases where the phenotype was not mentioned (not present or not evaluated). A detailed description of the statistical methodology can be found in Supporting Information 2.

## 3 Results

**FIGURE 1:**
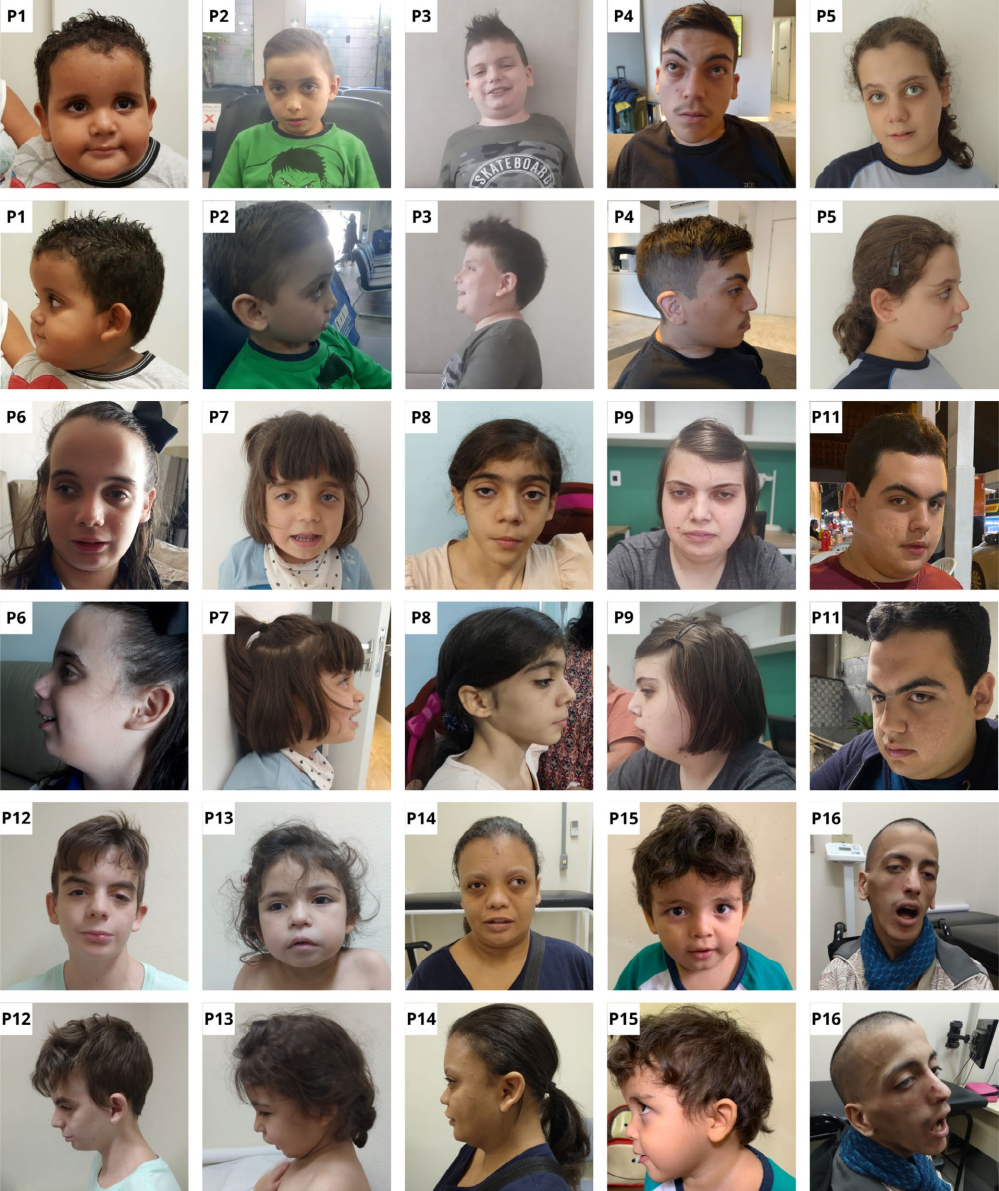
Craniofacial characteristics of 15 of the 16 individuals with XGS, emphasizing the varied nonspecific nature of facial dysmorphisms in this condition.

Sixteen individuals with XGS were evaluated in this study. Figure 1 shows photographs of 15 of these patients, highlighting the presence of nonspecific facial dysmorphisms. Clinical evaluation revealed a constellation of features consistent with XGS, including ID (100%), hypotonia (100%), absent speech or delayed speech development (93.7%), ASD (86.7%), and nonspecific facial dysmorphisms (100%). The detailed clinical characterization and information can be found in Supporting Information 3. Three additional clinical manifestations not previously associated with XGS were observed: hyperphagia (68.8%), shortening of the Achilles tendon (53.3%), and gonadal/genital anomalies (87.5% of males). Additionally, developmental regression was documented in four patients.

All 16 detected *AHDC1* pathogenic variants are classified as loss of function (four nonsense and 12 frameshift), presumptively leading to the production of a truncated protein. Only four of them had previously been reported in the literature; the 12 novel variants were deposited in ClinVar.

**FIGURE 2:**
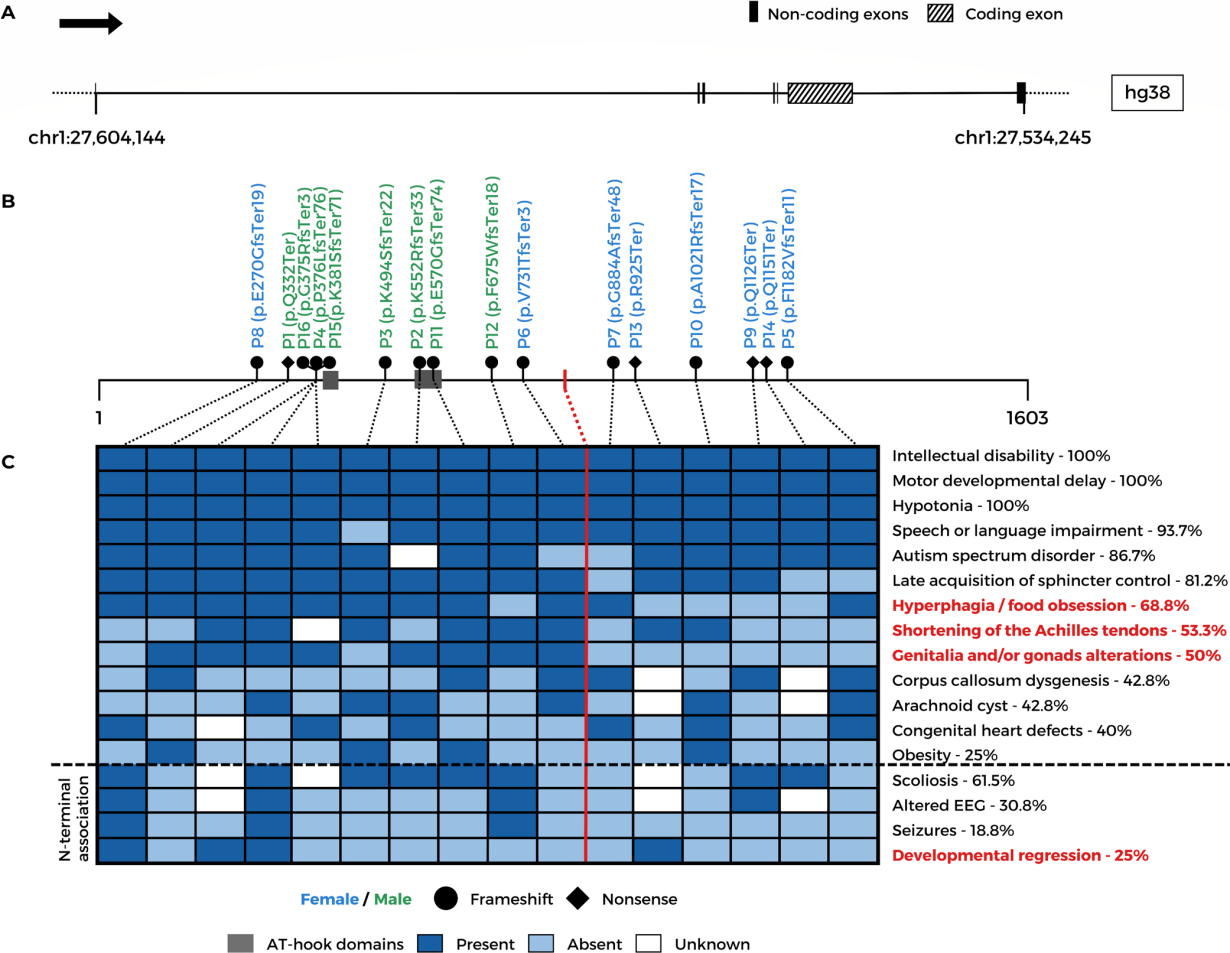
*AHDC1* pathogenic variants and major clinical characteristics of 16 individuals with XGS. (A) Schematic representation of the *AHDC1* gene (NM_001029882.3); the black arrow indicates the direction of transcription, and the striped rectangle represents the single coding exon (sixth), while the black vertical lines or rectangles are noncoding exons. (B) Positions of the 16 *AHDC1* variants through the gibbin protein (total length of 1603 amino acids), with both AT‐hook domains (according to the UniProt database) indicated by dark gray boxes. Variants identified in male or female patients are in green or blue, respectively; filled circles represent frameshift variants, whereas nonsense variants are represented by diamond‐shaped symbols. (C) Description of the observed phenotypic traits; each column represents a patient, and lines are phenotypes. Novel phenotypes reported in the present study are depicted in red. Dark blue and light blue rectangles indicate presence or absence, respectively, of a given phenotype; white rectangles denote unknown/unavailable information. The red vertical line marks the middle of the protein and separates the phenotypes observed in patients with variants in the first half of the protein from those with variants in the second half. The four phenotypes below the dashed line are reported to be associated with N‐terminal variants.

The major clinical and molecular data of the patients are summarized in Figure 2. The genomic position of the causative variants varies along the single coding exon (sixth, NM_001029882.3) of the *AHDC1* gene (Figure 2A). Six out of 16 patients carried variants mapped to the first third of the gibbin protein (N‐terminal), 7/16 were in the second third (middle), and 3/16 were in the last (C‐terminal) (Figure 2B).

Statistical analysis was performed with a total of 50 patients (16 from the present report and 34 from Khayat et al. [7]). Table 1 and Supporting Information 4 present data and statistical results. We found that the possible frequency values for scoliosis and epilepsy among patients with N‐terminal variants were 0.26–0.54 (average value 0.34) and 0.28–0.56 (average value 0.36), respectively. For C‐terminal carriers, the respective frequency values are 0.08–0.17 (average value of 0.10) and 0.10–0.23 (average value of 0.12). Therefore, for both phenotypes, their frequency among N‐terminal variant carriers is approximately three times greater than among C‐terminal carriers.

**TABLE 1:** Frequencies of scoliosis and epilepsy among reported cases of individuals with Xia–Gibbs syndrome carrying N‐terminal or C‐terminal *AHDC1* variants.^a^

		X	Y	Z	N	$p_{1}=X/N$	$p_{2}=X/\left(N-Z\right)$	p
Nter	Scoliosis	13	11	26	50	13/50 = 0.26	13/24 = 0.54	0.34
Cter		4	20	26	50	4/50 = 0.08	4/24 = 0.17	0.10
Nter	Epilepsy	14	11	25	50	14/50 = 0.28	14/25 = 0.56	0.36
Cter		5	17	28	50	5/50 = 0.10	5/22 = 0.23	0.12

^a^ The total number of individuals carrying N‐terminal (Nter, amino acids 1–801) and C‐terminal (Cter, amino acids 802–1603) variants that present a given phenotype is represented by X; Y indicates that the phenotype is absent. Z represents the cases in which the phenotype was not mentioned, either being absent (frequency given by $p_{1}$) or not investigated ($p_{2}$). p is the mean estimated frequency for the upper and lower proportion limits $p_{1}$ and $p_{2}$.

**FIGURE 3:**
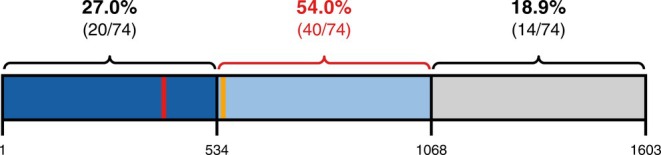
Distribution of causal variants of XGS cases reported in the literature considering their amino acid positions in the gibbin protein. The gibbin protein (total length of 1603 amino acids) is divided into three parts, with the approximate percentage and absolute number of *AHDC1* pathogenic variants mapped to each region according to a literature review (95 XGS cases). The dark blue, light blue, and light gray rectangles represent the N‐terminal, middle, and C‐terminal thirds of gibbin, respectively. The vertical lines represent the two AT‐hook domains, according to the UniProt database; the first (red) extends from amino acid 396 to 408, whereas the second (orange) extends from amino acids 544 to 556.

**FIGURE 4:**
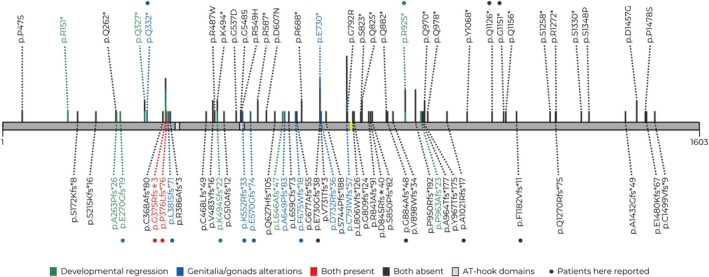
Pathogenic variants in the gibbin protein identified in XGS cases and report of developmental regression and/or genitalia/gonad alterations. The gray horizontal bar represents the gibbin protein (1603 amino acids), with its two AT‐hook domains (according to the UniProt database; light gray). The vertical yellow line marks the middle of the protein. The vertical bars indicate the position of the *AHDC1* variants in the protein; the stacked vertical bars proportionally represent the number of cases reported for each variant. Green, blue, and red indicate cases with developmental regression, gonadal/genital alterations, or both, respectively. Dark gray vertical bars represent patients without either phenotype. The patients described in this study are indicated by dots.

We reviewed the pathogenic/likely pathogenic variants reported in the scientific literature for XGS cases (*n* = 95), with 74 different variants mapped to gibbin (Figure 3). Supporting Information 5 describes the cDNA and protein nomenclatures, type, and reference for each *AHDC1* variant. Figure 4 depicts the position of their *AHDC1* variants in the gibbin, highlighting those with descriptions of developmental regression and/or genitalia/gonad alterations.

**FIGURE 5:**
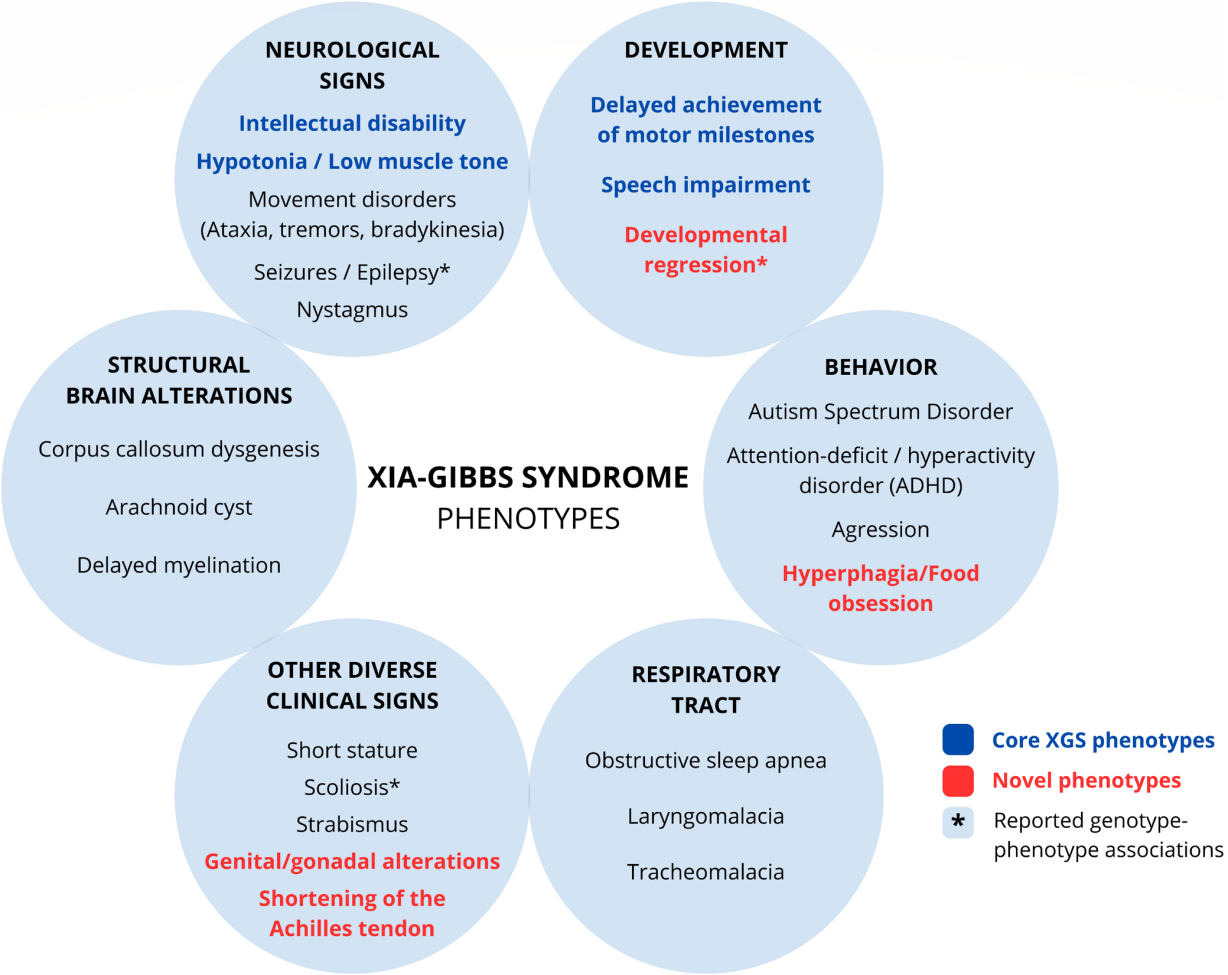
Summary of the XGS clinical signs. Core phenotypes are highlighted in blue, while novel phenotypes identified in this study are shown in red. Asterisks mark clinical signs with reported phenotype associations with N‐terminal variants.

In summary, Figure 5 presents the core phenotypes of XGS, a heterogeneous group of additional clinical features reported in patients, documented genotype–phenotype associations, and novel phenotypes observed in the present cohort.

## 4 Discussion

The clinical pattern revealed in this study underscores XGS heterogeneity. The core clinical signs of XGS [20] ‐ ID, hypotonia, delayed achievement of motor milestones, and speech impairment ‐ are exhibited by almost every patient. However, several other clinical features were present in each patient, and this highly variable expressivity might indicate that additional genetic or epigenetic factors are required to define the spectrum or severity of manifestations, together with other hit variants and/or environmental influences. Although all patients presented with ID, the degree of cognitive impairment and dependence on daily activities varied. There are no typical clinical characteristics suggestive of the syndrome, including the heterogeneous facial features they present. Speech disorders, including lack of speech, speech acquisition delay, and dysarthria, were present in all but one patient (P3). Overall, brain abnormalities were found in 12/14 patients with previous brain imaging exams. Corpus callosum dysgenesis was observed in 6/14 patients, as well as arachnoid cysts, both of which are clinical signs consistently reported in XGS [1]. Other structural brain alterations less frequently reported were enlarged cisterna magna, temporal and occipital polymicrogyria, and hypomyelination, among others. Macrocephaly was present in two patients (P1 and P2) and has been previously described in a few other cases [5, 16, 21].

Several anomalies in foot morphology were also documented (13/15) in our cohort, including pes cavus, which has been associated with XGS in other published cases [2, 3, 5, 9, 21, 22, 23, 24].

Behavior disturbances suggestive of ASD were present in 13/15 patients; however, only seven of them were formally diagnosed with ASD, and 7/16 patients experienced aggressive behavior and/or self‐aggression episodes. One interesting finding is that cannabidiol treatment for one of the patients (P4), who showed hyperactivity, ASD, and episodes of aggressive behavior, had a positive outcome.

Previously, XGS was linked to short stature [1, 25, 26]. In our cohort, the height of most patients was within normal values for age and sex. Only four patients (P8, P14, P15, and P16) had measurements at least two standard deviations below the age–sex mean; however, two were above average (P5 and P6). Additionally, seven patients presented ophthalmological alterations. Among them, six exhibit strabismus (P4, P5, P10, P14, P15, and P16), which is common among XGS patients [25]. Other eye anomalies and vision issues are described in Supporting Information 3.

Two genotype–phenotype associations previously suggested by Khayat and colleagues [7] were also checked in our group of patients. First, they reported an association between *AHDC1* variants truncating the N‐terminal region and seizures [20]. In the present study, electroencephalogram alterations—absence episodes without seizures, myoclonic epilepsy, and severe seizures—were present in 4/12 patients; however, patient P9 never experienced seizures. All three patients with seizures carried variants mapped to the N‐terminal region of the gibbin protein, reinforcing this reported association. However, the other seven patients who carried variants in this region of the protein did not exhibit this clinical sign, clearly indicating incomplete penetrance of this feature.

Another previously reported association with the first half of the protein was the presence of scoliosis [20]. Most of our patients who exhibited scoliosis also carried variants within the first half of gibbin (6/8); the two exceptions (P9 and P14) carried variants near the C‐terminal end. Therefore, our data support a strong, although incomplete, association between this phenotype and the genomic position of *AHDC1* variants. Importantly, the presence of hypotonia did not necessarily coexist with scoliosis in our cohort. Combining our findings with data reported by Khayat and colleagues [7], the results from statistical analysis strongly indicate that, among patients carrying N‐terminal variants, the frequency of any of the two analyzed phenotypes (scoliosis and epilepsy) is approximately three times greater than that of C‐terminal patients.

Notably, developmental regression was observed in four patients. P4, P8, and P13 presented with progressive loss of speech and communication skills, whereas P4 also lost other cognitive abilities. Motor retrogression was observed in P8 and P16. This phenotypic trait has already been described in seven other XGS cases [3, 5, 6, 12, 21, 22, 27], encompassing different ages of onset and levels of impairment. One of them has been linked to the recurrence of seizures, which might be the case for P4, and especially P8, with myoclonic epilepsy; however, P13 and P16 never experienced seizures. A recent report associates the advent of developmental regression with the progression of ASD‐related signs and with reduced social interaction due to the Covid‐19 pandemic [27]. Therefore, according to the literature and our data, 9/11 cases with regression carry variants mapped to the N‐terminal third of the protein, varying from amino acids 151 to 646. The two exceptions carry variants within the second third (amino acids 925 and 963, Figure 4). Thus, the occurrence of regression also can potentially be associated with variants in the N‐terminal region of gibbin.

It is important to highlight that Jiang et al. [14], whose work proposed three distinct clinical subtypes of XGS, did not identify a significant genotype–phenotype correlation between phenotypic classes and variant position. This discrepancy between the findings of Jiang et al. and those of our study, as well as previous studies, may be attributed to methodological differences. Both our study and that of Khayat et al. [7] evaluated individual phenotypes, whereas Jiang et al. [14] employed a multinomial logistic regression approach. Furthermore, we emphasize that additional genetic and environmental factors beyond variant type and position are likely critical for phenotype determination in XGS, potentially explaining the divergent clinical traits observed in individuals carrying the same variant.

The wild‐type gibbin protein is localized in the cell nucleus. Based on heterologous expression of mutated gibbin labeled with fluorescent tags in HeLa cells, Khayat and colleagues [7] reported that the truncated protein at position 262 was in the cytoplasm, whereas the isoforms that were truncated at other positions (791, 925, 970, and 1270) were detected only in the nucleus [20]. The different subcellular localizations may have implications for genotype–phenotype associations, but further functional studies are still needed to evaluate this hypothesis and unravel the critical points for phenotypic changes, especially in cell models derived from patients.

The collection of variants from the literature shows that the vast majority are frameshift/nonsense variants, with missense variants reported in only 11 cases, corresponding to 10 different variants. Although there is an increased number of variants in the middle of the protein (between amino acids 556 and 1080, as shown in Figure 3), no clear hotspots are observed. The last third of the protein, however, exhibits a smaller number of reported variants. Given previous associations between more severe phenotypes in carriers of N‐terminal variants in gibbin, it is possible that individuals with C‐terminal variants exhibit milder phenotypes, which may be underrepresented in exome sequencing cohorts due to lower clinical suspicion and delayed diagnosis. Therefore, we hypothesized that the reduced number of variants observed in the last third of the protein may reflect a detection bias.

Our data revealed three novel phenotypes related to the clinical picture of XGS, which were not previously reported: hyperphagia/food obsession, shortening of the Achilles tendon, and gonadal/genital anomalies.

Hyperphagia/food obsession was consistently observed in 11/16 patients, most of whom carried variants within the first half of the gibbin protein. With respect to patients' eating behavior, their parents reported food anxiety associated with fast eating, either by putting a large amount of food in their mouths or by swallowing too fast, usually not chewing enough (P4, P5, P7, and P16); constant search for food (P5, P6, P7, P8, and P15); ingestion of inedible matter (PICA—raw flour—P7; soap, clay, and insects—P8); and increased salivation (P5, P11, and P16); however, some patients presented food selectivity (P2, P6, P9, and P12). High and constant interest in culinary‐related videos/TV shows (P7 and P16) and playing with food‐related activities (P5, P7, and P8) were also observed. Notably, three of the patients (P1, P4, and P16) underwent previous genetic investigations for Prader–Willi syndrome (OMIM#176270), which is characterized by hyperphagia, obesity, and other clinical signs [26]. In addition, P11 was initially suspected to present Kabuki syndrome (OMIM#147920), a condition previously referred to in the literature as a syndromic form of obesity, although the current evidence does not firmly establish obesity as a central characteristic of this syndrome [28].

However, only 4/11 patients with hyperphagia/food obsession have obesity (Figure 2C and Supporting Information 3). Importantly, patients who have obesity also present milder cognitive and motor impairments and are less dependent on daily activities, such as eating by themselves. Patients who are not obese also have more limitations, as they usually rely on their parents/caretakers for feeding; although they exhibit a constant search for food and an increased appetite, the ingestion of food is far more controlled. Interestingly, P4 had obesity, but experienced substantial weight loss and improvement in his eating behavior once he started receiving cannabidiol treatment. Additionally, 3/4 of patients with obesity take risperidone, a medication that can stimulate hyperphagia and/or weight gain (P1, P3, and P11). Although P16 also takes risperidone, he is not able to walk and has not developed obesity, further contributing to our hypothesis.

Notably, the Xia–Gibbs Society website (xia‐gibbs.org) includes a section dedicated to introducing the patients, and most reported activities were related to food. Interestingly, in a recent study, Li and colleagues [29] established an *Ahdc1*± mouse strain that exhibited obesity, hyperleptinemia, insulin resistance, abnormal glycolipid metabolism, and hepatic steatosis, which suggest that *Ahdc1* may be a key regulator of obesity and energy metabolism. However, no change in food intake was observed in *Ahdc1* ^+/‐^ mice.

Shortening of the Achilles tendon was identified in 8/15 patients, with different degrees of severity, including elongation surgery, which was needed in some patients. The occurrence of tendon shortening may be associated with reduced stimulation of the lower limbs in the first years of life due to delayed motor development, but this phenomenon remains to be evaluated. Furthermore, other connective tissue alterations have been reported in XGS patients, including joint hypermobility [9], which was observed in P2, P4, P12, and P13. Recently, the gibbin protein was reported to be associated with early epithelial development via the regulation of mesoderm maturation, elucidating some XGS phenotypes [30].

Notably, our XGS case series has a considerable frequency of genitalia and/or gonad alterations, observed in 8/16 patients (7/8 males); these alterations include unilateral (P12) and bilateral (P4, P15, and P16) cryptorchidism, hypogenitalism (P1, P6, P11, and P16), and testicle enlargement (P2). Only three reported cases in the literature included information on genitalia and/or gonad development (all males) [2, 3, 31], and the AHDC1 variant positions of these patients are highlighted in Figure 4. Considering the observed frequency of these characteristics here, it is probably underdocumented in the literature. Additionally, the greater ease of verifying gonadal alterations in males than in females may interfere with documenting these alterations in general.

The identification of novel associated phenotypes contributes to better delineation of the full spectrum of XGS. In addition, early recognition of specific phenotypes allows for anticipated care, tailored interventions, and guidance for families, ideally through an individualized approach given the marked clinical variability in XGS. The establishment of structured routines, environmental regulation, and psychological support are potential strategies for managing a hyperphagic behavior, especially considering the possible psychological implications of satiety dysregulation and dietary restriction. Furthermore, since the shortening of the Achilles tendon can lead to progressive functional limitations, ultimately requiring surgical correction, regular orthopedic surveillance and physiotherapy are crucial. Finally, cryptorchidism increases the risk of clinical complications, such as testicular torsion and malignancy; hence, incorporating urological evaluations into the clinical management of male SXG patients is recommended.

Developmental regression is a challenging feature, particularly in individuals with atypical development. Documenting motor and cognitive abilities is essential to elucidate whether the regression is either an intrinsic feature of the syndrome or only the result of potentially manageable secondary factors, such as epilepsy. Moreover, we emphasize the importance of electroencephalogram assessments in the neurological monitoring of XGS patients, as seizure episodes can lack obvious motor manifestations and might go unnoticed, especially in individuals with communication impairments.

The pathophysiological mechanisms underlying *AHDC1* pathogenic variants, as well as the precise functions of gibbin [32], require further clarification in order to provide new perspectives for future treatments. Unveiling pathogenic mechanisms in XGS is crucial for the development of targeted therapeutic strategies, such as protein replacement, inhibition of activated pathways, or epigenetic modulation. Therefore, functional studies are vital resources for translating genetic knowledge into clinical outcomes.

The identification of XGS molecular markers could be important for reclassifying variants of uncertain significance (VUS), especially in patients with *AHDC1* missense variants. Recently, we developed the first cellular (iPSC—induced pluripotent stem cells) and animal (zebrafish) models to study XGS [33], shortly after which two publications reported other iPSC [34] and mouse models [29]. Future functional studies could help unravel the complex phenotypic variability and explore potential ways to modulate the phenotypes discussed in this manuscript, with the goal of developing therapies to increase the quality of life of XGS patients.

## 5 Conclusions

In summary, our findings provide insights into the clinical and molecular characteristics of XGS in 16 Brazilian patients, emphasizing the phenotypic variability and genetic complexity of this rare neurodevelopmental disorder. Recognition of the expanded phenotypic features, with three characteristics that had not been previously documented (hyperphagia/food obsession, shortening of the Achilles tendon, and genitalia/gonad alterations), and identification of novel pathogenic variants contribute to improved diagnosis, management, and genetic counseling for affected individuals and their families. Additionally, we corroborate the association between causal variants in the first half of the *AHDC1* coding region and the manifestations of epilepsy and scoliosis, as well as suggest a novel association with developmental regression.

## Author Contributions

Ana Cristina Victorino Krepischi, Laura Machado Lara Carvalho and Carla Rosenberg conceived and designed the study. Maísa Ganz Sanchez Sennes was responsible for the initial writing of the manuscript and produced the figures with the guidance of Ana Cristina Victorino Krepischi and Laura Machado Lara Carvalho. Laura Machado Lara Carvalho and Maísa Ganz Sanchez Sennes recruited patients, collected and analyzed clinical data, and obtained biological samples. Chong Ae Kim, Matheus Augusto Araújo Castro, Maria Isabel Melaragno, and Giovana Manilli Toccoli contributed to patient recruitment and obtaining clinical and molecular data. Matheus Augusto Araújo Castro, Salmo Raskin, Juan Clinton Llerena Júnior, Débora Romeo Bertola, Eny Maria Goloni Bertollo, Erika Cristina Pavarino, Larissa Sampaio de Athayde, Cecilia Barbosa Buck, Maria Betânia Pereira Toralles, Chong Ae Kim, Gustavo Marquezani Spolador, Paulo Alberto Otto, Caroline Brandão Piai, Fernando Kok and Ceres Schmitz Cechella clinically evaluated the patients. Maísa Ganz Sanchez Sennes, Davi Mendes Campo Fialho, and Sofia Oliveira Farias performed Sanger sequencing to verify the presence and segregation of *AHDC1* variants. Mariluce Riegel‐Giugliani contributed to obtaining biological samples. Paulo Alberto Otto performed the statistical analysis. All authors have read, edited, and approved the final manuscript.

## Ethics Statement

Approval for the study was obtained from the Research Ethics Committee of the Institute of Biosciences of University of São Paulo (CAAE 80921117.5.00000.5464). The procedures used in this study adhere to the tenets of the Declaration of Helsinki.

## Consent

Informed consents to participate in the study and publish photographs were obtained from the patients' parents.

## Conflicts of Interest

The authors declare no conflicts of interest.

## Peer Review

The peer review history for this article is available at https://www.webofscience.com/api/gateway/wos/peer‐review/10.1111/cge.14777.

## Supporting information

Supporting Information 1.

Supporting Information 2.

Supporting Information 3.

Supporting Information 4.

Supporting Information 5.

## Data Availability

Data on the novel genetic variants reported in this study are openly available in ClinVar (https://www.ncbi.nlm.nih.gov/clinvar/): P1‐SCV002060418; P2‐SCV004042692; P3‐SCV004042693; P4‐SCV004042694; P5‐SCV004042695; P6‐SCV001135230.1; P7‐SCV004042696; P8‐SCV004042702; P9‐SCV004042703; P10‐SCV004042704; P12‐SCV005201021; P14‐SCV005201023.
