# Supplementary material for: Clinical and Molecular Characterization of Xia–Gibbs Syndrome: Expanding the Phenotypic Spectrum in a Brazilian Cohort
Source: Clin Genet. 2025 Jun 11;108(6):654–63. doi: 10.1111/cge.14777 (PMC12580479; doi:10.1111/cge.14777)
Supplement: Supplementary file 1 — Supporting Information 1. [file CGE-108-654-s002.docx]

**Supplementary Material 1.** Semi-Structured Interview Script For The Families Of Xia-Gibbs Syndrome Patients

Date of birth:

Age at diagnosis:

Family history: Does the patient have siblings? How many? What is their biological sex? Are they all from the same parents? Is there any case of intellectual disability or developmental delay in the family?

Other genetic exams performed:

Gestational and birth history (gestational period, fetal movements, any complications, type of delivery, crying at birth, etc.):

Neonatal history: what was the APGAR score? Did the patient have jaundice? Was the suction effective? Were there any feeding problems? Did they exhibit hypotonia?

When and how did you first notice differences in your child's development?

Motor development milestones: Age at which your child could support their head, sit without support, crawl and walk.

Speech development: Age at which your child said their first words and started to form sentences. Have any health professionals mentioned that their speech development was delayed? How is their speech articulation currently? Is there any report from a speech therapist?

Is there any report from a neurologist?

When did your child start school? How has their development been at school? What grade are they in? Are they able to read, write, do basic math, and recognize numbers and colors? Are they able to focus? How is their interaction with classmates? How was the school's reception and support? What information has the school provided about your child’s development and behavior?

Does your child respond well to commands/instructions?

Can your child deal with money?

In which types of tasks and activities are they dependent on others? And independent?

Have they ever undergone an IQ test with a psychologist?

How is their interaction with the family?

Behavior: Do they exhibit repetitive movements, ritualistic behaviors and/or hyperfocus on any activity? Do they show aggressiveness towards others or themselves? Do they show affection? Are they anxious or restless?

Do you have any report provided by a psychologist?

What are their eating habits? Do they have any restrictions? Do they show anxiety around food? Do they constantly search for food?

Approximate weight and height measurements:

If your child exhibits short stature, have they undergone growth hormone tests?

Has any alteration in the gonads or genitalia been reported?

How is their motor coordination?

Are they able to control sphincters?

Do they have scoliosis? Have you consulted an orthopedist? Is there a spinal radiography report?

Have they undergone bone age tests?

Do they have a history of recurring infections?

How is your child’s sleep? Do they snore? Do they have sleep apnea?

Have they ever had seizures? With or without fever? Have they done an electroencephalogram exam?

Have they done any brain imaging exam? Are there any abnormalities?

Do they use any medication?

Is there any other health information you would like to share?

**References:**

CARDOSO-DOS-SANTOS, Augusto César; SILVA, Thiago Christiano; FACCINI, Anderson Silveira; *et al*. Novel *AHDC1* Gene Mutation in a Brazilian Individual: Implications of Xia-Gibbs Syndrome. **Molecular Syndromology**, v. 11, n. 1, p. 24–29, 2020.

CARVALHO, Laura Machado Lara. **Investigação Genética Da Obesidade Sindrômica E Desenvolvimento De Modelos Biológicos Para Estudo Da Síndrome De Xia-Gibbs**. Tese De Doutorado, Instituto de Biociências da Universidade de São Paulo, 2023. Disponível em:<https://www.teses.usp.br/teses/disponiveis/41/41131/tde-23032023-174554/pt-br.php>. Acesso em: 11 maio 2023.

DÍAZ-ORDOÑEZ, Lorena; RAMIREZ-MONTAÑO, Diana; CANDELO, Estephania; *et al*. Syndromic Intellectual Disability Caused by a Novel Truncating Variant in AHDC1: A Case Report. **Iranian Journal of Medical Sciences**, v. 44, n. 3, p. 257–261, 2019.

GOYAL, Chanan; NAQVI, Waqar ; SAHU, Arti. Xia-Gibbs Syndrome: A Rare Case Report of a Male Child and Insight into Physiotherapy Management. **Cureus**, v. 12, n. 8, 2020.

MURDOCK, David R.; JIANG, Yunyun; WANGLER, Michael; *et al*. Xia–Gibbs syndrome in adulthood: a case report with insight into the natural history of the condition. **Molecular Case Studies**, v. 5, n. 3, p. a003608, 2019.
