# Supplementary material for: Clinical and Molecular Characterization of Xia–Gibbs Syndrome: Expanding the Phenotypic Spectrum in a Brazilian Cohort
Source: Clin Genet. 2025 Jun 11;108(6):654–63. doi: 10.1111/cge.14777 (PMC12580479; doi:10.1111/cge.14777)
Supplement: Supplementary file 2 — Supporting Information 2. [file CGE-108-654-s001.docx]

# Supplementary Material 2. Statistical analysis

In many papers, as in the present report, a series of signs/symptoms is collected from a set of patients pre-examined without the application of a disease-specific, standard guide of anamnesis/physical examination and/or in a series of cases collected from the literature, resulting that sometimes a cardinal sign/symptom is not mentioned in the description of a given affected individual. This could have happened for two reasons: (a) in some cases, the sign/symptom was not mentioned because it was not present; (b) in other cases, however, it was not mentioned because the sign/symptom was not investigated.

If X, Y, and Z (with X + Y + Z = N) are the observed numbers of a given sign or symptom described respectively as present (X) or absent (Y), or non-mentioned (Z), in a series of N cases collected as described above, under the hypothesis (a) that the non-mentioned characteristic was absent, the estimate for its frequency is given by p_1_ = X/N, with expected binomial variance var(p_1_)= p_1_(1-p_1_)/N; under the mutually exclusive hypothesis (b) that the non-mentioned sign/symptom was not investigated, its frequency estimate is given by p_2_ = X/(N-Z), with expected binomial variance var(p_2_) = p_2_(1-p_2_)/(N-Z). The true estimate of the frequency of the sign/symptom is therefore an unknown quantity within an interval with lower and upper limits given exactly by p_1_ and p_2_. An approximate but reliable estimate of the mean frequency p can be obtained by weighing the estimates p_1_ and p_2_ by the reciprocals of their expected binomial variances. The method described above was used for the first time in the paper by **Pardono et al. (2003).**

In order to compare the frequencies of three important signs/symptoms among patients carrying N-terminal (N-ter) and C-terminal (C-ter) variants, data from the present paper and those described by Khayat et al. (2021) were aggregated. The total number of patients (16 from the present report and 34 from Khayat et. al) was N = 50. Table 1 lists the results obtained, together with the quantities (X, Y and Z) used to estimate p_1_ and p_2_ in the two different conditions (scoliosis and epilepsy) in carriers of N and C terminal variants.

Despite the fact that a standard statistical method cannot be applied to test our data, the possible frequency values of patients with scoliosis are contained in the interval 0.26 - 0.54 (average value 0.34) among N-ter patients and within the interval 0.08 - 0.17 (average value 0.10) among C-ter patients. Among patients with epilepsy, the corresponding values for carriers of N-ter and C-ter variants are 0.28 - 0.56 (average value 0.36) and 0.10-0.23 (average value 0.12).

These results (Table 1) strongly indicate that among patients carrying N-ter variants the frequency of any out of the two conditions analyzed (scoliosis and epilepsy) is three times increased approximately in relation to Cter patients.

**TABLE 1.**

For an explanation of the symbols used on this table, please read the above text.

|  |  | **X** | **Y** | **Z** | **N** | **p_1_ = X/N** | **p_2_ = X/(N-Z)** | **p** |
| --- | --- | --- | --- | --- | --- | --- | --- | --- |
| **N-ter** | **scol** | **13** | **11** | **26** | **50** | **13/50 = 0.26** | **13/24 = 0.54** | **0.34** |
| **C-ter** |  | **4** | **20** | **26** | **50** | **4/50 = 0.08** | **4/24 = 0.17** | **0.10** |
| **N-ter** | **epil** | **14** | **11** | **25** | **50** | **14/50 = 0.28** | **14/25 = 0.56** | **0.36** |
| **C-ter** |  | **5** | **17** | **28** | **50** | **5/50 = 0.10** | **5/22 = 0.23** | **0.12** |

Pardono E, van Bever E, van den Ende J, Havrenne PC, Iughetti P, Maestrelli SRP, Costa F O, Richieri-Costa A, Frota-Pessoa O, Otto PA. Waardenburg syndrome: Clinical differentiation between types I and II. **Amer. J. Med. Genet. 117A: 223-235, 2003.** PMID:12599185 WOS:000182401100004
