# Supplementary material for: Clinical and Molecular Characterization of Xia–Gibbs Syndrome: Expanding the Phenotypic Spectrum in a Brazilian Cohort
Source: Clin Genet. 2025 Jun 11;108(6):654–63. doi: 10.1111/cge.14777 (PMC12580479; doi:10.1111/cge.14777)
Supplement: Supplementary file 4 — Supporting Information 4. [file CGE-108-654-s005.docx]

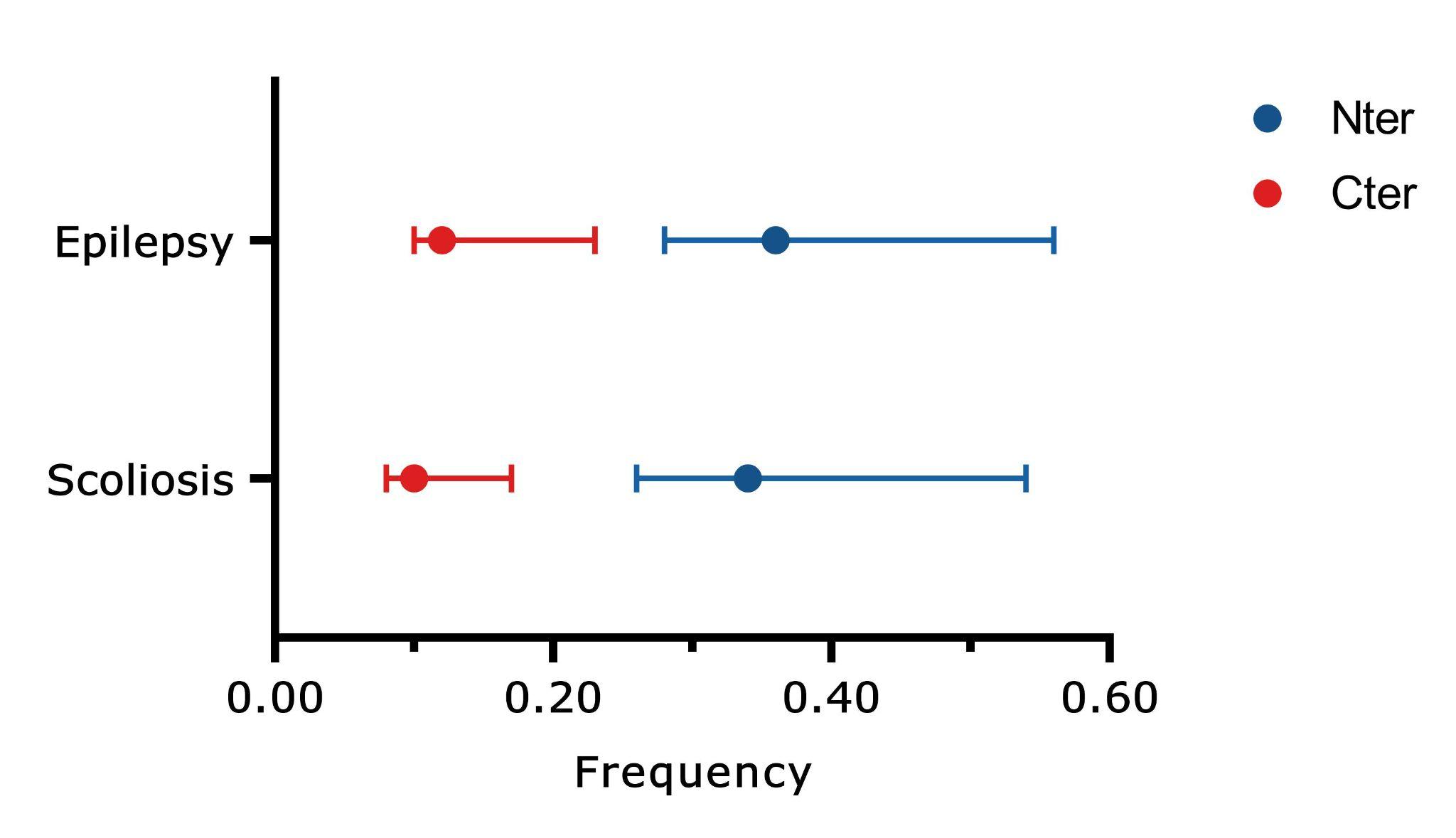


**Supplementary Material 4.** Exact possible limits of the frequencies of scoliosis and epilepsy among patients with N-terminal and C-terminal variants.

Patients with N-terminal and C-terminal variants are represented in blue and red, respectively. The dots indicate their corresponding average values, calculated taking into account the samples size and variance of the binomial limit intervals.
